# Long-Term Analysis of Suicide Incidence Among Patients with Lung Cancer: A Population-Based Longitudinal Study

**DOI:** 10.3390/jcm14124070

**Published:** 2025-06-09

**Authors:** Eunjoo Kim, Wongi Woo, Sungsoo Lee, Hee-Taik Kang

**Affiliations:** 1 Department of Psychology, Gangnam Severance Hospital, Yonsei University College of Medicine, Seoul 06273, Republic of Korea; ejkim96@yuhs.ac; 2 Department of Internal Medicine, Dignity Health St. Joseph’s Medical Center Stockton, Stockton, CA 95204, USA; 1keywoo@gmail.com; 3 Department of Thoracic and Cardiovascular Surgery, Gangnam Severance Hospital, Yonsei University College of Medicine, Seoul 06273, Republic of Korea; 4 Department of Family Medicine, Severance Hospital, Yonsei University College of Medicine, Seoul 03722, Republic of Korea

**Keywords:** suicide, lung cancer, cancer survivor, mental health, national registry

## Abstract

**Objectives:** Patients with cancer often experience severe mental distress, and suicide is an important issue, particularly prevalent in individuals with lung cancer. The present study aimed to investigate the longitudinal incidence of suicide among patients with lung cancer using national registry data. **Methods:** A population-based retrospective review of patients diagnosed with lung cancer in 2008 was conducted. Longitudinal medical records, including clinical outcomes and medical insurance data, were investigated. The primary outcome was the incidence of suicide, compared between patients undergoing the first curative treatment option (surgery or non-surgery). Cox proportional hazard regression models were used to adjust for medical history, sociodemographic variables, and lifestyle factors. **Results**: Among the 4495 patients included, 1306 (29.1%) underwent surgery as the first treatment. Compared to the non-surgery group, the surgery group was younger and had a lower Charlon comorbidity score (*p* < 0.001), higher physical activity (*p* < 0.001), and higher income level (*p* < 0.001). The total number of suicides was 28 (0.62%). The surgery group demonstrated similar trends in the development of suicide and early 5-year follow-up to those of the non-surgery group. **Conclusions:** The longitudinal risk of suicide among patients with lung cancer increased. Both surgical and non-surgical treatment groups demonstrated similar suicide trends, although patients in the surgery group had multiple protective factors.

## 1. Introduction

Lung cancer is the leading cause of cancer-related morbidity worldwide; about 2.5 million people globally were diagnosed with it in 2022 [1,2]. Lung cancer remains the leading cause of cancer death globally, largely due to its late diagnosis—often at stage III or IV in over 70% of patients—which results in a low five-year survival rate of 28.1% [3]. Lung cancer is also the leading cause of cancer-related deaths in South Korea [4]. To address the burden of lung cancer, significant advancements, including tyrosine kinase inhibitors [5], immunotherapy [6], sublobar resection [7], and multidisciplinary care, have enhanced the quality of care and treatment outcomes. Although these developments have significantly improved long-term survival rates, patients with lung cancer still face unmet needs, such as quality of life and mental health issues [8,9].

Depression and suicide after a cancer diagnosis have become important issues with the growing population of cancer survivors. Compared to the general population, patients with cancer experience increased mental distress attributed to cancer symptoms, immunologic or endocrinologic disturbances, adverse effects from treatments, and anxiety arising from their diagnosis [10]. Psychiatric conditions can negatively affect various medical outcomes, such as perioperative complications, extended hospital stays, higher rates of readmission, and increased risk of postoperative suicidal ideation. Therefore, improved management of psychiatric symptoms could potentially enhance patient outcomes, affecting quality of life and treatment adherence, ultimately influencing survival. In addition, there are non-cancer-related causes in cancer patients such as cardiovascular disease, cerebrovascular disease, pneumonia and influenza, and suicide/self-inflicted injury [11], attributed to the aging population and early diagnosis of cancer, resulting in decreased cancer-specific mortality [4,11]. Therefore, several academic societies have suggested guidelines for vigilantly monitoring patients with cancer for mental distress and suicide prevention [12,13,14].

Among all types of cancers, lung cancer appears to have a higher likelihood of depression or suicide after diagnosis [15,16]. Specifically, individuals with lung cancer exhibit significantly higher rates of suicide (standardized mortality ratio [SMR] 2.04–13.4) within the first year after diagnosis [16,17]. Although several studies have investigated the incidence and risk factors among patients with lung cancer, most had limited data with short-term (less than 3 years) follow-up and small sample sizes [18,19]. Moreover, studies from the Surveillance, Epidemiology, and End Results (SEER) database have intrinsic limitations, as they do not represent the entire population [20,21,22]. These studies also often fail to adjust for preexisting psychiatric disorders or other potential confounders, such as information on income level, alcohol or smoking abuse, other medical comorbidities, and lifestyle factors.

Therefore, the present study aimed to investigate the long-term incidence of suicide after lung cancer diagnosis and identify high-risk groups. We also compared suicide incidence according to the therapeutic approach: surgery versus non-surgery.

## 2. Materials and Methods

### 2.1. Data Sources and Study Population

This retrospective study used data from the Korean National Health Insurance Database (NHID). The Korean National Health Insurance is operated by the Korean National Health Insurance Service (NHIS) and is the only insurance to which almost all Koreans must subscribe. The Korean NHIS collects insurance information, medical utilization (outpatient clinic visits and hospitalization), and medical records, including prescriptions, procedures, diagnostic codes, medical costs, and data from national health screening programs. In addition, death information based on death certificates from Statistics Korea was merged with the NHID. The national health screening programs are regularly (usually biennially) provided for adults aged 40 years or older or with relevant conditions such as viral hepatitis infection or liver cirrhosis. National health screening programs collect anthropometric data, blood pressure, lifestyle factors (cigarette smoking, alcohol consumption, and physical activity), personal medical history, and laboratory data, including blood glucose, liver enzymes, and lipid profiles. This information is saved to the Korean NHID and provided for research purposes by the NHIS.

**Figure 1:**
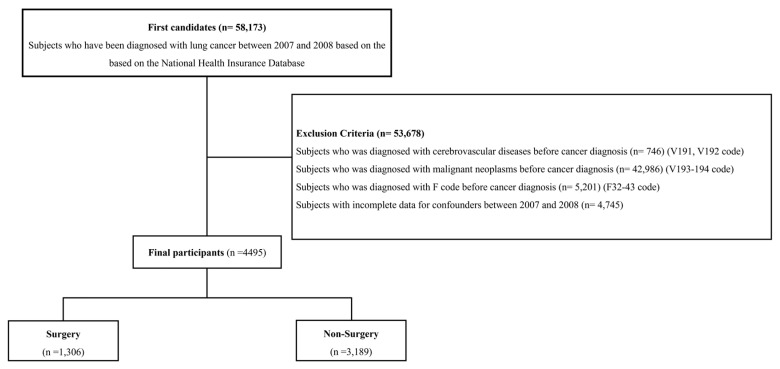
Study cohort selection process.

Figure 1 presents the inclusion and exclusion criteria in this study. Initially, patients diagnosed with any type of cancer between 2007 and 2008 were selected based on the NHID (n = 761,380). Of these patients, those diagnosed with lung cancer were included based on the NHID (n = 58,173). Among them, those who met at least one of the following criteria were excluded: (1) diagnosed with cardiocerebrovascular diseases before enrollment (n = 746); (2) diagnosed with any malignant neoplasms before enrollment (n = 42,986); (3) diagnosed with the F code (F32–43) before enrollment (n = 5201); and (4) those with incomplete data for confounders between 2007 and 2008 (n = 4745). Finally, 4495 patients with malignant lung neoplasms were included.

This study was conducted in accordance with the Declaration of Helsinki and approved by the Institutional Review Board of Gangnam Severance Hospital, Yonsei University Health System (IRB No. 2-2023-0358).

### 2.2. Definition of Cancer, Suicide, and Covariates

Cancer was defined using special codes (V193 and V194). Each cancer type was classified based on the main diagnostic code (C00−C99). The Korean NHIS grants special codes to patients with rare, intractable, or severe diseases. Special diseases, such as cancer or cardiocerebrovascular diseases, were encoded as V codes and strictly monitored because patients with these diseases pay much less out-of-pocket for hospital bills than patients without them. Among these special codes, V193 and V194 are malignant neoplasms. When V193 or V194 is combined with C codes based on the International Classification of Diseases 10th Revision (ICD-10), the occurrence of malignant neoplasms can be determined. Individuals with C33−C34 (or C73) based on ICD-10 and V193–V194 between 2007 and 2008 were included in this study.

The primary outcome was the incidence of suicide. Death by suicide was defined based on death certificates. Patients were categorized into surgery and non-surgery groups. Surgery groups were identified using the following codes: wedge resection, O1401; Segmentectomy, O1410; Lobectomy, O1421; Bilobectomy, O1422; Pneumonectomy, O1431.

The study date was defined as the day when a patient was encoded in V193–V194 with C33−C34 (lung cancer). If suicide or death occurred as the primary outcome, the end date was the earlier date of these events. If death or suicide does not occur, the end date was determined as the later date between the last outpatient clinic visitation or participation in the last national health screening program.

Body mass index (BMI; kg/m^2^) was calculated by dividing the body weight (kg) by height (m) squared. Cigarette smoking was categorized into three groups: (1) never smokers, individuals who had never smoked cigarettes; (2) former smokers, individuals who smoked cigarettes in the past but had quit smoking; and (3) current smokers, individuals who currently smoke cigarettes. Alcohol consumption was classified as follows: rare drinkers, who drank alcohol less than once a week; moderate drinkers, who drank alcohol once to twice a week; and heavy drinkers, who drank alcohol three or more times per week. Physical activity was divided into three groups: (1) low, engaged in physical activity less than once a week; (2) moderate, engaged in physical activity one to four times a week; and (3) high, engaged in physical activity five or more times per week. Economic status was stratified into three groups based on self-reported monthly household income: low, 0–30th percentile; middle, 31st–70th percentile; and high, 71st–100th percentile.

The Charlson Comorbidity Index (CCI) was developed to categorize patient comorbidities based on ICD diagnostic codes reported in the administrative data. Each comorbidity had a weighted score ranging from 1 to 6 based on the adjusted risk of death or associated medical resource utilization. The sum of all weighted scores indicated a single comorbidity score for each patient. A score of zero indicates no comorbidities, whereas higher CCI scores demonstrate a greater likelihood that the anticipated health outcomes will result in death or higher medical utilization [23]. CCI accurately predicts health outcomes such as death in hospitalized older adult patients, those with advanced renal diseases, and patients who underwent surgery [24,25,26].

### 2.3. Statistical Analysis

All variables are presented as mean ± standard deviation for continuous variables and as number of patients (percentage) for categorical variables. To compare the means or percentages between the two groups, independent *t*-tests for continuous variables and chi-square tests for categorical variables were used. The cumulative incidence of the primary outcome (death from suicide) was estimated using the Kaplan–Meier method and compared using log-rank tests. To investigate whether therapeutic modalities (surgery versus non-surgery) increased the risk of suicide, Cox proportional hazards regression models were built and adjusted for age, cigarette smoking, alcohol consumption, physical activity, household income, and CCI.

Statistical significance was set at two-sided *p* < 0.05. The SAS Enterprise Guide version 7.1 was used as the statistical package (SAS Inc., Cary, NC, USA), and R studio version 3.3.3 was used to perform the statistical analyses.

## 3. Results

### 3.1. Baseline Characteristics of Study Populations

Among 4495 patients with lung cancer included in this study, 1306/4495 (29.1%) underwent surgical treatment at the year of diagnosis. Table 1 presents the baseline characteristics of the study participants according to treatment type. There were significant differences (*p* < 0.001) in age, sex, and BMI between the surgery and non-surgery groups. The non-surgery group had older patients, higher systolic blood pressure/fasting glucose levels, and higher CCI scores. Regarding lifestyle factors, the non-surgical group had higher alcohol consumption levels, were former/current smokers, and had lower physical activity levels. Most patients with low income were in the non-surgical group (Table 1).

### 3.2. Incidence of Depression/Suicide Between Two Groups

**Figure 2:**
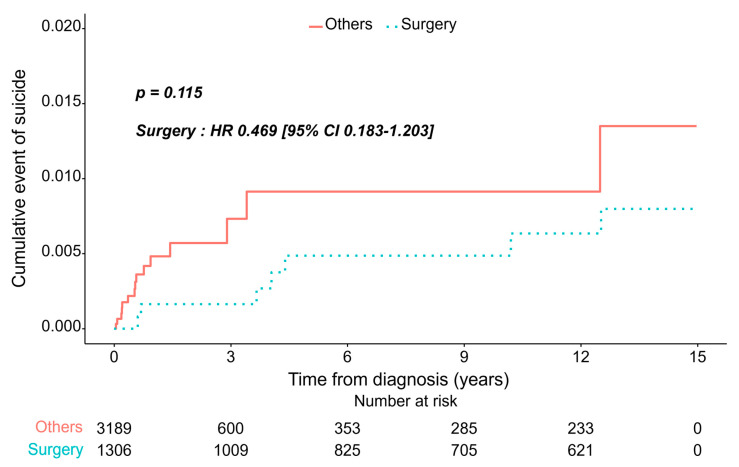
Cumulative incidence of suicide according to treatment modalities.

**Figure 3:**
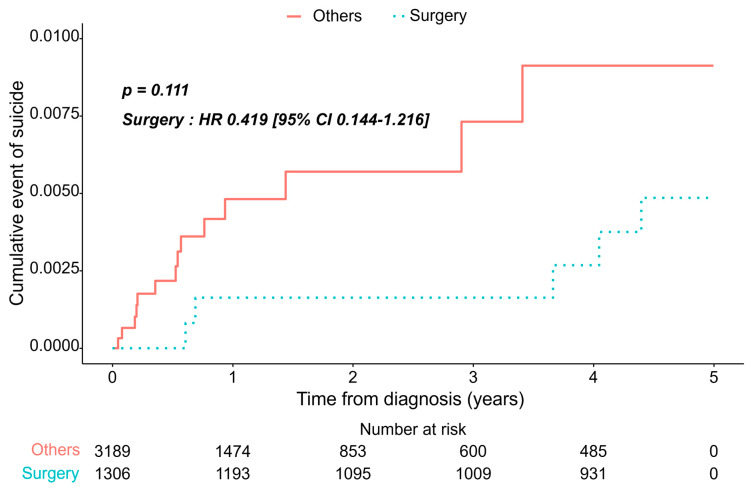
Cumulative incidence of suicide according to treatment modalities within 5 years of diagnosis.

The non-surgical group had a higher proportion of patients who committed suicide during the mean follow-up period. However, Kaplan–Meier analysis with a log-rank test revealed that the cumulative incidence of suicide between the two groups and the non-surgical and surgical groups had similar incidences of suicide: hazards ratio (HR) 0.469 (95% confidence interval [CI] 0.183–1.203, *p* = 0.115; Figure 2). A similar pattern was observed within the early 5-year follow-up (HR, 0.419; 95% CI 0.144–1.216, *p* = 0.111; Figure 3).

### 3.3. Risk Model Prediction of Suicide

Table 2 presents the Cox proportional hazards regression models for suicide prediction between the two groups. Three models that modulated relevant clinical factors revealed no difference in terms of the long- or short-term occurrence of suicide according to treatment type.

## 4. Discussion

To the best of our knowledge, the present study represents the first longitudinal analysis of suicide among patients with lung cancer based on a nationwide registry that incorporates comprehensive demographic, social, and clinical factors. The strength of our study is that the complete cause-of-death (suicide) determination was based on the death certificate from Statistics Korea, representative national data coverage that minimizes biases caused by missing data, and the ability to adjust for various sociodemographic factors, preexisting psychiatric disorders, and other potential confounders, such as lifestyle factors or comorbid medical conditions, owing to the availability of relevant data in the NHID of Korea. Notably, the incidence of suicide was not significantly different according to treatment modality (surgery versus non-surgery). Although there was a slightly increased incidence of suicide in the early period after diagnosis in the non-surgical treatment group, the difference was not clinically significant and was similar between the two treatments in the long term.

In general, the non-surgical groups tend to have a considerably higher risk of mortality, as surgery is usually indicated for early-stage or locally advanced lesions with resectability [27]. As indicated in our data, the non-surgical treatment group had more risk factors than the surgery group, including older age, comorbidities presented as CCI, and lifestyle-related risk factors. Notably, these factors are known to contribute to suicide. However, the non-surgery and surgery groups had similar suicide rates; this result could be attributed to either elevated levels of mental distress in the surgery group or relatively preserved stress-handling skills in patients with advanced lung cancer.

Despite the earlier cancer stage in patients who underwent surgery for lung cancer, cancer surgery itself could lead to a more extensive deterioration in the quality of life. Surgery can cause a more noticeable impact in terms of day-to-day functions [28,29], cosmetic changes [30], and extensive distress [31]. It can also cause and lead to post-traumatic syndrome-related symptoms [32,33]. Rauma et al. reported that patients with resectable lung cancer continue to experience permanently decreased quality of life after surgery [34]. While the specific reasons for these findings are not clear, it is speculated that the psychological consequences and the subsequent adjustment reaction caused by a cancer diagnosis and primary treatment are significant risk factors for emotional distress [11]. In patients with breast cancer, anxiety and depression are similarly observed in patients with early and advanced stages [35]. Early psychiatric intervention during the post-operative period could provide greater benefits to patients [36] and reduce the incidence of chronic pain and related psychiatric distress [37].

In the present study, the non-surgical group tended to have more patients with unresectable tumors, such as stage III or IV. The advanced stage is a significant prognostic factor for clinical outcomes, and patients’ perceptions of their stages are critical for cancer survivorship. This can aggravate mental stress and increase the risk of suicide. In other cancers, such as prostate and GI cancers, patients exhibit significantly lower rates of depression during the early stage of the disease [38]. Notably, there was no significant difference in the suicide rate between the two groups in our study. Given the higher mortality rates and low expected survival of lung cancer, perceptions among patients might be similar among patients regardless of stage or treatment. This finding aligns with those of previous studies, indicating that the risk of suicide is linked to cancers with low survival rates and limited treatment options. Potter et al. reported that patients who underwent surgery for cancers with lower 5-year survival rates died by suicide earlier than those with less aggressive cancers, highlighting the need to screen patients with cancer for psychiatric conditions and suicide risk [15].

The prediction of suicide among patients with lung cancer is important. Zhang et al. suggested a model from the SEER database [20], where patients who refused or were medically intolerable for surgery, and underwent chemotherapy or radiotherapy, had a high risk for suicide. Undergoing therapy after cancer diagnosis offered greater comfort or confidence in the chance of recovery, potentially alleviating some distress induced by cancer [20]. In other studies, risk factors for suicide among patients with lung cancer included late stage, early period after diagnosis [16], and pre-treatment mental distress [39]. Owing to limited clinical data in our cohort and a relatively low number of patients who committed suicide, validating these findings was challenging. However, future studies using a nationwide registry on this topic could identify patients who are at risk of suicide.

This study investigated current monitoring strategies for patients with lung cancer after surgery. Physicians and surgeons tend to consider mental health issues to be less important in patients with early or resectable disease. Consequently, there has been insufficient effort to advocate for patients’ mental health problems in these specific groups. Monitoring and postoperative examinations typically focus on detecting recurrence or other cardiovascular events, with limited evidence supporting the use of depression screening tools or psychiatric interventions. Therefore, our study could raise awareness among clinicians about the need for a more comprehensive assessment of cancer survivorship [9].

Multimodal prehabilitation came up recently regarding the care for cancer patients, and it includes multidisciplinary medical care, exercise, nutrition, and psychological support [10,40]. Due to its complexity and heterogenous clinical scenarios, the introduction of multimodal prehabilitation has been limited. However, newly published studies demonstrated superior outcomes in cancer patients who had undergone surgery for lung, esophageal, colorectal, and ovarian cancers [40,41,42,43]. As our data exhibited unmet needs of patients in terms of psychological perspective, this type of multimodal approach would be beneficial in the long term. Further standardization of this measure is anticipated in the near future.

Our study has several limitations. First, several important variables such as histology, stage, and ECOG functional performance status, were not available in the NHIS. These factors should be integrated for objective interpretation. Among patients who underwent surgery, some might have undergone a non-curative operation for biopsy; therefore, including them with stage I patients who underwent surgery might have skewed the interpretation. Second, this study did not represent evolving lung cancer diagnosis and therapeutics. Significant improvements in lung cancer treatments have been achieved over the last 10 years, and chemotherapy and immunotherapy have dramatically improved; however, data on therapeutics were limited in our study. Therefore, our findings may not fully apply to current lung cancer cohorts. Nevertheless, our study underscores the enduring psychiatric burden of patients with lung cancer. Third, patients in Korea belong to a relatively homogeneous ethnic group and do not represent different ethnic diversity. The suicide rate in the general population may differ by nation. In addition, healthcare accessibility, in terms of social determinants of health, is notably higher in South Korea. Therefore, the implications of these results should be applied to each institution considering the availability and accessibility of mental health care.

## 5. Conclusions

This study demonstrates that patients with lung cancer have a higher incidence of suicide, regardless of treatment modality. Patients who underwent surgery exhibited a lower suicide rate at the beginning, but the difference was not significant after long-term follow-up. Further studies are required to evaluate the preventive effects of psychiatric interventions after a lung cancer diagnosis. This study emphasizes the need for a similar comprehensive approach for patients with lung cancer, regardless of their resectability.

## Figures and Tables

**Table 1:** Baseline characteristics of the study population.

	Surgical Resection	Others	*p*-Value
Clinical Variables	(n = 1306)	(n = 3189)	
Age, years	63.4 (±9.1)	68.0 (±10.1)	<0.001
<50	103 (7.9%)	177 (5.6%)	
50–59	305 (23.3%)	455 (14.3%)	
60–69	548 (42.0%)	942 (29.5%)	
≥70	350 (26.8%)	1615 (50.6%)	
BMI, kg/m^2^	23.5 (±3.0)	22.9 (±3.1)	<0.001
Systolic Blood pressure, mmHg	126 (±16)	129 (±17)	<0.001
Fasting blood sugar, mg/dL	101.4 (±28.1)	103.7 (±35.6)	0.034
Total cholesterol, mg/dL	192.5 (±35.8)	190.3 (±39.2)	0.126
SGPT, U/L	24.0 (±16.8)	23.8 (±19.3)	0.538
Charlson comorbidity index			<0.001
2	350 (26.8%)	562 (17.6%)	
3 or more	956 (73.2%)	2627 (82.4%)	
*Life-style related factors*			
Alcohol consumption			<0.001
Rare	97 (7.4%)	90 (2.8%)	
Moderate	992 (76.0%)	2506 (78.6%)	
Heavy	217 (16.6%)	593 (18.6%)	
Smoking history			<0.001
Never smoker	692 (53.0%)	1523 (47.8%)	
Former smoker	218 (16.7%)	455 (14.3%)	
Current smoker	396 (30.3%)	1211 (38.0%)	
Physical activity			<0.001
Low	24 (1.8%)	30 (0.9%)	
Moderate	1057 (80.9%)	2784 (87.3%)	
High	225 (17.2%)	375 (11.8%)	
Income level			<0.001
Low (0–6)	246 (18.8%)	676 (21.2%)	
Middle (7–13)	360 (27.6%)	1020 (32.0%)	
High (13–20)	700 (53.6%)	1493 (46.8%)	
Suicide			0.035
No	1303 (99.8%)	3164 (99.2%)	
Yes	3 (0.2%)	25 (0.8%)	

Data are presents as n (%) or mean ± standard deviation.

**Table 2:** Cox proportional hazards regression models for suicide according to treatment options.

	Surgery	Others	*p*-Value
	(n = 1306)	(n = 3189)	
**Model1: adjusted for age, sex**			
Hazard ratio (95% CI)	0.469 (0.183–1.203)	1 (Ref.)	0.115
**Model 2: adjusted for BMI, SBP, Total cholesterol, Fasting blood glucose, SGOT, in addition to variables in Model 1**			
Hazard ratio (95% CI)	0.481 (0.184–1.256)	1 (Ref.)	0.135
**Model 3: CCI, alcohol consumption, smoking history, physical activity, income levels, and depression history, in addition to variables in Model 2**			
Hazard ratio (95% CI)	0.489 (0.185–1.291)	1 (Ref.)	0.222

BMI, body mass index; CCI, Charlson comorbidity index; CI, confidence interval; SBP, systolic blood pressure; SGOT, serum glutamic-oxaloacetic transaminase.

## Data Availability

The data supporting this study are available from the corresponding author upon reasonable request.
